# Developing Clinical Phenotype Data Collection Standards for Research in Africa

**DOI:** 10.1155/2023/6693323

**Published:** 2023-09-19

**Authors:** Lyndon Zass, Katherine Johnston, Alia Benkahla, Melek Chaouch, Judit Kumuthini, Fouzia Radouani, Liberata Alexander Mwita, Nihad Alsayed, Taryn Allie, Dassen Sathan, Upendo Masamu, Milaine Sergine Seuneu Tchamga, Tsaone Tamuhla, Chaimae Samtal, Victoria Nembaware, Zoe Gill, Samah Ahmed, Yosr Hamdi, Faisal Fadlelmola, Nicki Tiffin, Nicola Mulder

**Affiliations:** ^1^Computational Biology Division, Department of Integrative Biomedical Sciences, IDM, University of Cape Town, Cape Town, South Africa; ^2^Laboratory of BioInformatics, BioMathematics and BioStatistics LR16IPT09, Institut Pasteur de Tunis, Tunis, Tunisia; ^3^South African National Bioinformatics Institute (SANBI), Life Sciences Building, University of Western Cape, Bellville, Cape Town, South Africa; ^4^Chlamydiae & Mycoplasmas Laboratory Research Department, Institut Pasteur du Maroc, 20360 Casablanca, Morocco; ^5^Muhimbili Sickle Cell Program, Department of Hematology and Blood Transfusion, Muhimbili University of Health and Allied Sciences, Dar-es-Salaam, Tanzania; ^6^Kush Centre for Genomics & Biomedical Informatics, Biotechnology Perspectives Organization, Khartoum 11111, Sudan; ^7^Software Information Systems Department, FOICDT, University of Mauritius, Reduit, Mauritius; ^8^Department of Mathematics and Physics, Cape Peninsula University of Technology, Bellville, Cape Town, South Africa; ^9^Laboratory of Biotechnology, Environment, Agri-Food and Health, Faculty of Sciences Dhar El Mahraz-Sidi Mohammed Ben Abdellah University, Fez 30000, Morocco; ^10^Division of Human Genetics, Department of Pathology, Faculty of Health Sciences, University of Cape Town, Cape Town, South Africa; ^11^Department of Molecular Biology, Johannes Gutenberg University, Mainz, Germany; ^12^Laboratory of Biomedical Genomics and Oncogenetics, Institut Pasteur de Tunis, University of Tunis El Manar, Tunis, Tunisia; ^13^Laboratory of Human and Experimental Pathology, Institut Pasteur de Tunis, Tunis, Tunisia; ^14^Wellcome Centre for Infectious Disease Research in Africa, Institute of Infectious Diseases and Molecular Medicine, Faculty of Cape Town, University of Cape Town, Cape Town, South Africa

## Abstract

Modern biomedical research is characterised by its high-throughput and interdisciplinary nature. Multiproject and consortium-based collaborations requiring meaningful analysis of multiple heterogeneous phenotypic datasets have become the norm; however, such analysis remains a challenge in many regions across the world. An increasing number of data harmonisation efforts are being undertaken by multistudy collaborations through either prospective standardised phenotype data collection or retrospective phenotype harmonisation. In this regard, the Phenotype Harmonisation Working Group (PHWG) of the Human Heredity and Health in Africa (H3Africa) consortium aimed to facilitate phenotype standardisation by both promoting the use of existing data collection standards (hosted by PhenX), adapting existing data collection standards for appropriate use in low- and middle-income regions such as Africa, and developing novel data collection standards where relevant gaps were identified. Ultimately, the PHWG produced 11 data collection kits, consisting of 82 protocols, 38 of which were existing protocols, 17 were adapted, and 27 were novel protocols. The data collection kits will facilitate phenotype standardisation and harmonisation not only in Africa but also across the larger research community. In addition, the PHWG aims to feed back adapted and novel protocols to existing reference platforms such as PhenX.

## 1. Introduction

Biomedical data are increasingly used in high-throughput and interdisciplinary approaches, with data generation and knowledge discovery occurring at a faster pace than ever before, particularly in the areas of genomics and bioinformatics [1, 2]. As a result, there has been an increase in multiproject, consortium-based collaborations and meta-analyses. Such efforts have the potential to expedite knowledge discovery in resource-limited research communities, particularly low- and middle-income countries, where funding for large sample collections is limited [3]. Advances in genomics research have raised the need to match large-scale phenotypic data which incorporate social, environmental, and clinical factors, with genetic information. However, researchers still struggle to collect complete and valuable data in a standard manner, which hinders data integration and collaborative efforts [4]. Meaningful analysis of multiple heterogeneous phenotypic datasets is difficult, and at times, impossible, without standardisation of the data elements prior to collection or retrospective harmonisation of the collected phenotypic data.

Phenotype standardisation involves harmonisation of the way researchers define and collect information about clinical phenotypes and environmental exposures. This is opposed to retrospective phenotype harmonisation, which involves the integration of phenotype data collected or defined in various ways and is often employed in meta-analyses and collaborative research efforts. Phenotype standardisation may be employed for various reasons. For example, to promote operability and the interoperability of data coming into a biomedical database, collaborative and multisite studies need to ensure that their collection methods and dataset formats are as similar as possible to reduce bias and assist with overall study management and quality control. One way to achieve this is by employing ontologies to standardise the definitions for trait and case classification as previously shown [5]. In doing so, phenotype standardisation can also improve the quality of research outputs, as has been shown in pharmacogenetics and kidney disease research [6–8]. More recently, the NIH-funded PhenX (consensus measures for Phenotypes and eXposures) Toolkit (https://www.phenxtoolkit.org/) has emerged as a leading resource for well-established standard protocols for the collection of phenotype and exposure information. This publicly available online catalogue contains standard and recommended protocols to extract the maximum value from data collected for genomics research, although these protocols are also applicable to other fields [9–11]. Measures hosted by PhenX have been successfully employed to identify opportunities for cross-study collaboration through established databases such as the database of Genotypes and Phenotypes (dbGaP) and the Cancer Data Standards Registry and Repository (caDSR) [12]. Despite these advances, some protocols within PhenX may lack regional validity since they were largely developed from a Western perspective. For example, protocols related to the concepts of diet, nuclear family, and extended family may need to be adapted since these vary across the world.

The Human Heredity and Health in Africa (H3Africa) consortium was established to drive novel and innovative genomics research in Africa and build capacity on the continent [13]. As part of the consortium, the H3Africa Bioinformatics Network (H3ABioNet) was formed to support these efforts, with a particular focus on the production and sharing of FAIR (Findable, Accessible, Interoperable, and Reproducible) data [14]. With the goal of facilitating the standardisation and interstudy sharing of phenotypic data within H3Africa and beyond, these initiatives established the H3Africa Phenotype Harmonisation Working Group (PHWG) in 2014. The PHWG aimed to build a core set of phenotypes to be collected within the consortium and develop protocols to facilitate the collection of these phenotypes. This work was later expanded to cover specific research or disease domains, which were represented in clusters across the consortium [15, 16]. Here we describe the development and dissemination of standard phenotype data collection kits for genomics research, which are adapted to ensure their suitability for use in African settings.

## 2. Methods

### 2.1. Core Phenotypes

The PHWG oversaw the establishment of a standard set of CORE PHENOTYPES, relevant to the study of the diseases or traits explored in H3Africa. Given the diversity of diseases and populations studied within H3Africa (covering the study of neonatal respiratory diseases to adult renal diseases to the transmission of zoonotic tuberculosis in rural pastoral populations), the initial set of phenotypes was extremely broad. The PHWG catalogued a set of CORE PHENOTYPES based on the number of H3Africa studies that measured them (each phenotype had to be measured in more than 3 studies). As a result, a set of 26 core phenotypes and 10 discretionary phenotypes were identified. These were selected based on their general interest and applicability in most study populations, relative ease of collection, and whether they were a primary phenotype in one of the H3Africa studies (to facilitate identification of common controls). To formulate these CORE PHENOTYPES, the working group chose to use the PhenX Toolkit to format the selected phenotypes, which initiated the development of the H3Africa Standard Case Report Form (Standard CRF). H3Africa grantees were encouraged, where possible, to measure the CORE PHENOTYPES in each research study so that the community could assess the power of the opportunity to measure a common set of phenotypes across a maximum number of projects. Following the initial development of the Standard CRF, the Working Group (WG) revised it to ensure its application to both paediatric and adult participants.

### 2.2. Domain-Specific Toolkits

**Figure 1:**
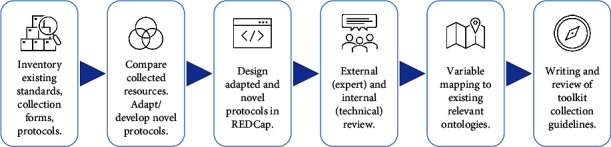
Development process of core phenotypes and domain-specific kits.

Building on the development of the H3Africa Standard CRF, the PHWG expanded the scope of development to cover domain-specific research group needs within H3Africa, to facilitate potential phenotype standardisation and harmonisation within and across these groups, should they wish to expand their collaborative efforts. As with the CORE PHENOTYPES, consensus mapping between H3Africa projects of a specific group was first conducted by cataloguing the domain-specific phenotypes within the groups and identifying common overlaps. Thereafter, phenotypes were mapped to PhenX to identify the appropriate protocols associated with the phenotypes. Where relevant protocols were identified but not suited to implementation in Africa, the protocols were adapted for such use. Where relevant protocols were not identified, novel protocols were developed. Thereafter, guidelines to facilitate data collection were established for both the Standard CRF and domain-specific kits. The overall development process for these two components is illustrated in Figure 1.

### 2.3. Technical Development

Since the platform was widely employed by H3Africa for clinical data collection, REDCap (Research Electronic Data Capture) was selected to develop the data collection kits. REDCap is a secure, web-based software platform designed to support data capture for research studies. It provides (1) an intuitive interface for validated data capture; (2) audit trails for tracking data handling and export procedures; (3) automated export procedures for seamless data downloads to common statistical packages; and (4) procedures for data integration and interoperability with external sources [17]. PhenX also provides protocols that generate REDCap.xml files, and the software itself lends itself to easy design and sharing of data collection instruments.

Before building the kits, some functional elements had to be considered in terms of the variables and project structure within REDCap:

1. Each domain-specific kit needed to be able to stand alone with the CORE PHENOTYPES or be incorporated with any other combination of kits. To this end, all kits were built in a single REDCap project to eliminate the possibility of duplicate variable names before splitting them into separate domain-specific kits. In addition, because several kits have branching logic based on participants' biological sex and age (collected in the CORE PHENOTYPES), each domain-specific kit was packaged alongside the CORE PHENOTYPES but also made available as separate data collection instruments for import.
2. Consistent coding for missing data must be applied across data collection kits; therefore, each module has a preset list of missing data codes, which can be modified by users.
3. Variable naming conventions needed to be useful to the end-user; therefore, variable names relevant to what was being collected were employed to avoid nonsensical indexed naming.
4. Consistent coding was an absolute necessity, so basic codes for common responses and formats were applied throughout.
5. Cosmetically, all the forms were formatted with the same style, and the basic REDCap forms were maintained, without the use of external kits. Field embedding was limited to a few fields necessary to accommodate mobile data collectors.

The REDCap instruments for every kit are intended to allow studies to include additional data elements and can be adapted to suit the needs of any study. The only caution for developers is to consider the elements already included as recommended for collection and, when making changes, to consider the branching logic already in place (this is primarily with respect to the display of age or biological sex questions).

### 2.4. Ontology Mapping

Once the data dictionaries were finalised, each variable in the data dictionaries was mapped to a relevant ontology code, where possible, to facilitate interoperability and reproducibility. Ontology mapping was conducted using the Ontology Lookup Service (OLS) and Zooma developed by the European Bioinformatics Institute (EBI). Domain-dedicated and well-maintained ontologies were preferred during this mapping. Ontologies were checked and revised to ensure complete correspondence of mapped variables, and the ontology code was incorporated into the data dictionary file for machine readability.

### 2.5. Review

The final set of data collection kits (CORE PHENOTYPES and Domain-specific Kits) underwent multiple rounds of review, both internal and external, to ensure quality and usability. The external review involved distributing a survey to African experts in a particular field to review the selected phenotypes and the collection protocols. The experts included in the survey comprised of researchers in leadership positions in a particular field and professionals involved in data collection and management. The survey allowed experts to review each element and associated protocol included in a toolkit, designate its applicability or lack thereof, and make additional recommendations to improve the toolkit. The internal review involved the revision of ontology mappings by a dedicated team, two rounds of quality and consistency checks using the established guidelines, and a review of the structure and organisation of the data dictionaries to ensure technical validity and usability.

## 3. Results

### 3.1. Data Collection Kits

**Figure 2:**
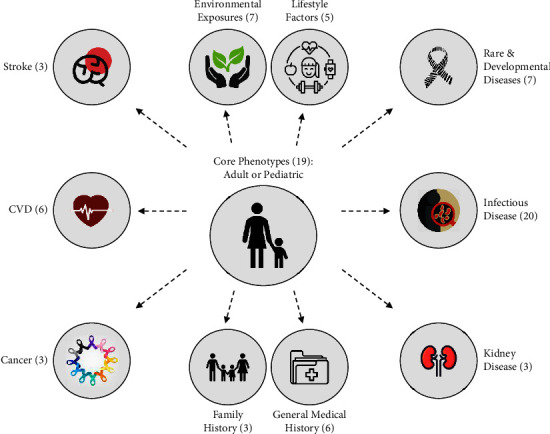
Overview of data collection kits developed by H3Africa. The number of protocols indicated per kit is given in brackets; CVD: cardiovascular disease.

In total, the PHWG produced 11 data collection kits, including the CORE PHENOTYPES and 10 domain-specific kits. All kits contain protocols adapted for use in both paediatric and adult participants. In total, they included 82 protocols, of which 38 were existing protocols (from PhenX or other existing initiatives, e.g., WHO, CIDRI-Africa, and the HIV Data Exchange Initiative), 17 were adapted, and 27 were novel protocols. Adapted protocols are protocols that are based on existing protocols but were edited (e.g., addition/removal of fields or rephrasing/restructuring of protocols) to improve their application in low- and middle-income regions and Africa, in particular. Examples of adapted protocols include those used to capture information on diet and household characteristics. Novel protocols are those for which it was not possible to identify appropriate existing protocols and were thus newly developed, such as asthma, pregnancy history, birth history, and family history protocols. An overview of the established data collection kits is illustrated in Figure 2 and summarised in Table 1.

**Figure 3:**
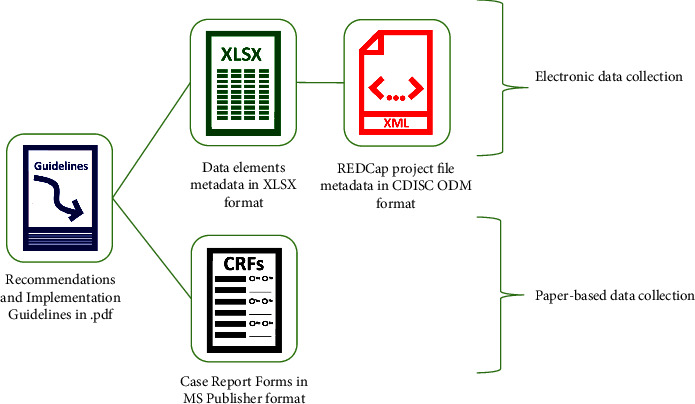
Data collection kit components.

As illustrated in Figure 3, each kit is composed of multiple components, including a REDCap implementation file, a data dictionary to be implemented on the platform of choice, a case report form or data collection document, and a guideline, to facilitate the use of the kit.

The CORE PHENOTYPES were first released internally within the H3Africa consortium in 2017/2018, then publicly thereafter. The additional domain-specific kits were finalised and released in 2022. All kits are currently available on various public platforms, including the H3ABioNet website (https://h3abionet.org/data-standards/phenotype-data-collection-standard), H3ABioNet GitHub (https://github.com/h3abionet/h3aphenstds), and ZivaHub/Figshare (https://zivahub.uct.ac.za/projects/H3ABioNet_H3Africa_Phenotype_Standards_Project/149305). Since its release on ZivaHub/Figshare, all related pages have been viewed for a total of 1795 times and the kits have been downloaded 288 times (as of May 2023).

### 3.2. External Review

In total, the data collection kits were reviewed by 45 individuals (experts) through a survey, with the majority of respondents coming from South Africa (16), Tunisia (5), and Nigeria (4). In addition, the majority of respondents were classified as non-H3Africa experts (17), while most of the H3Africa respondents were associated with the Phenotype Harmonisation (15), Cardiovascular Disease, and Rare Diseases H3Africa WGs. The majority of respondents identified themselves as experts in rare and developmental disorders (13), infectious diseases (13), and family history (12).  Figure S1 (A and B) shows the complete breakdown of survey respondents.

### 3.3. External Use

The CORE PHENOTYPES have been disseminated internally for an extensive period of time (since 2018) and, to our knowledge, have been implemented by at least 9 H3Africa projects, mainly the projects initiated in the second round of H3Africa funding. These include studies involving exome sequencing and genome-wide association and epigenetic studies conducted on study populations from South Africa, Nigeria, Rwanda, Ethiopia, and Botswana and focused on the genetic underpinnings of infectious diseases and cardiovascular, mental, and developmental disorders [18–23]. The CORE PHENOTYPES served as a point of reference to facilitate retrospective phenotype harmonisation by the Cardiovascular H3Africa Innovation Resource (CHAIR) [24] and the Cardiometabolic Disorders in African-Ancestry Populations (CARDINAL) initiatives and to generate synthetic phenotype data by the Common Infrastructure for National Cohorts in Europe, Canada, and Africa (CINECA) initiative [25]. Since the majority of the Domain-specific Kits were only finalised and disseminated in 2022, we do not yet have reports on validation for these kits, although, to our knowledge, the Genome Tunisia Collaborative Alliance has used a slightly modified and translated version of the CORE PHENOTYPES and Family History kits.

## 4. Discussion

In this report, we have described the efforts of the H3Africa PHWG to establish standard data collection protocols for relevant biomedical and health research in Africa and in resource-limited regions globally. As far as we are aware, this is the first such standardisation effort at the African level. It has been carried out within the framework of the H3Africa projects with a vision to enable African researchers to access and use them for newly developed projects, thus facilitating future data integration, phenotype harmonisation, and meta-analyses efforts, while also building a solid foundation for future collaborative efforts, both within the continent but also cross-continentally. We encourage use and feedback throughout the continent, to ensure continuous improvement on the existing recommendations.

As indicated, the established protocols not only encourage and promote standard data collection from the ground up but may also be useful in integrating phenotypic data from various sources retrospectively, i.e., harmonising phenotypes. Indeed, the kits are based on commonly collected phenotypes from specific research domains. When developing the protocols, the PHWG sought to find a balance between simplicity and completeness, and to cover various scenarios. The first step in many retrospective phenotype harmonisation efforts is typically identifying overlapping or common phenotypes across various projects and then to develop a data dictionary structured to allow transformation of data from different sources into a harmonised data structure [26]. In so doing, the data collection kits, and specifically standard data dictionaries, can facilitate two of the key steps in phenotype harmonisation.

The data collection kits were developed and released in adherence to FAIR principles and also promote FAIR principles in research projects which employ them. The kits were released on multiple platforms, including the H3ABioNet website, GitHub, and ZivaHub/Figshare, making them freely accessible to any interested user and allocating them unique identifiers to facilitate their findability. In addition, the kits provide a REDCap-based XML file which can be implemented directly on REDCap, which is broadly available for academic use, along with an EXCEL file which facilitates viewing for novice users and enables users to transform the file for the data management platform of their choice. The kits encourage interoperability and reproducibility in that they are standard recommendations based on existing standards and, additionally, employ ontology codes for machine-readable interoperability. They are also released with associated guidelines for reproducibility in practice.

Regarding the assessment of the validity of data collection kits, we had limited opportunity to measure the validity of the domain-specific kits in practice within the scope of H3Africa, given the time of development and final release of these kits. To counter this, we sought to assess and ensure the validity of the kits in other ways; therefore, we encouraged collaboration and worked alongside field experts to develop each of the kits, including, as previously illustrated, the Kidney Disease and Stroke kits [15, 16]. We also sought additional external feedback on the elements and data collection protocols included in the kits by surveying field experts. Finally, where existing standard protocols were found and applicable, like protocols observed in PhenX, we encouraged the use of these standards, which have previously undergone broad validity assessments. This was to avoid the development of new but overlapping protocols. Despite these measures, we recognise that the kits may have practical limitations not considered during the development and thus encourage feedback on the kits from the user community. Feedback may be provided through multiple channels, including submitting issues on GitHub and the H3ABioNet Helpdesk [27]. These also serve as an important channel through which we can track implementation of the kits. Although we encourage citation of kits when employed, many projects may not reference data collection forms in their methods section, representing a tracking barrier.

In contrast to the domain-specific kits, the CORE PHENOTYPES were released to H3Africa for a much longer period. H3Africa grantees were encouraged, where possible, to use the Standard CRF, as a guide to collect a common set of phenotypes to be measured in each research participant, although it was recognised that this was not always realistic, particularly because (1) there are costs in terms of time and effort to collect additional phenotypes, (2) recipients are not funded to collect phenotypes other than those in the original grant application, (3) some phenotypes have little or no relevance to certain populations and collection settings, and (4) some grants had already finalised their CRFs or were already recruiting subjects, which limits the possibilities for adding or revising measures. Despite the abovementioned considerations, several H3Africa projects did implement the CORE PHENOTYPES within their research projects and data collection processes, particularly the projects which were funded during H3Africa's second cycle (once the CORE PHENOTYPES were already established and released). Feedback from these projects was extremely positive regarding simplifying the CRF design process, the simplicity of use, and the comprehensive nature of design. As a by-product of collecting the CORE PHENOTYPES, various groups found great collaborative potential with regard to simple retrospective phenotype harmonisation, including the Mental Health and Cardiovascular Disease working groups, the latter of which developed a database for phenotypes harmonised to many of the CORE PHENOTYPES [24].

Although the kits have been successfully implemented by numerous studies, we recognise that existing barriers may prevent the more extensive implementation of the data collection kits. One such barrier is a lack of technical capacity to implement the kits electronically. This is one of the main reasons why the kits also include a CRF which promotes paper-based collection, the other being that research studies in low-income settings may often still rely on paper-based collection. To address such capacity gaps, we also aim to provide training materials exhibiting implementation of the kits on technical platforms. The other foreseeable barrier is that research initiatives based on existing cohorts are often hesitant to switch data collection methods once data collection has previously been conducted in a different manner, as this presents data integration issues downstream. To address this barrier, we need to also promote the kits as tools for retrospective phenotype harmonisation, as it has previously been used for by the Mental Health and Cardiovascular Disease working groups.

In the future, the primary efforts of the PHWG will be focused on promoting the use of the developed data collection kits on a broader scale, particularly in Africa. This may be achieved through endorsement by local research and funding bodies but also endorsement from previous users. In addition, the PHWG will be maintaining the data collection kits so that they remain relevant for future. One of the key goals for the project will be to feed back the adapted and novel protocols developed to PhenX for incorporation into the catalogue. In this manner, a broader set of users may be reached through an established body, which will, ultimately, facilitate the broad goals of the project. In addition, we will also investigate integration and interoperability with existing common data models such as the Observational Medical Outcomes Partnership (OMOP) model to increase broad usability [28].

## 5. Conclusion

The PHWG has successfully developed a series of easy-to-use data collection kits that cover a range of biomedical research fields. These kits should facilitate phenotype standardisation and harmonisation efforts on the African continent and the larger user community. The standards can form the basis for data models for post hoc data harmonisation. In addition to the abovementioned benefits, the data collection kits will also promote FAIR data principles, ultimately enabling data integration and interoperability. Finally, as mentioned previously, several novel data collection protocols were developed during these efforts, where relevant gaps were identified. These, along with protocols which were adapted from already established protocols, will be submitted for (re)incorporation into the PhenX platform.

## 6. Resource Availability

The resources discussed in the publication can be found on multiple platforms as listed below:

1. Website (https://www.h3abionet.org/component/sppagebuilder/page/259)
2. GitHub (https://github.com/h3abionet/h3aphenstds)
3. Figshare (https://zivahub.uct.ac.za/projects/H3ABioNet_H3Africa_Phenotype_Standards_Project/149305)

## Figures and Tables

**Table 1:** Overview of structure of data collection toolkits developed by H3Africa.

Toolkit	Protocols				
Core phenotypes	Demographics^+^	Smoking exposure^±^	Drug use^#^	Dyslipidemia^#^	CVD^∗^ Core^#^
	Anthropometrics^+^	Smoking status^#^	Medication log^+^	Cancer core^+^	HIV core^#^
	Blood pressure^#^	Alcohol exposure^−^	Diabetes history^+^	Kidney disease core^#^	Infectious disease core^+^
	Urine results^#^	Alcohol use^#^	Asthma^−^	Stroke history core^#^	

Environmental factors	Household characteristics^+^	Sanitation^+^	Occupational exposures^#^	Personal care products^#^	
	Water resource^+^	Occupational history^#^	Air contaminants^#^		

Lifestyle factors	Physical activity^#^	Dietary supplements^#^	Diet^+^	Caffeine intake^#^	Sleep habits^#^

General medical history	Vaccinations^#^	Current pregnancy status^±^	Last pregnancy outcome^±^		
	Allergies^+^	Pregnancy history^±^	Medical history^±^		

Family history	Family composition^±^	Family history^±^	Family history of traumatic life events^±^		

Cardiovascular disease	Angina^#^	Congestive heart failure^#^	Cardiac blood flow measurements^±^		
	Heart attack^#^	Thyroid disease^#^	Cardiovascular disease blood biomarkers^±^		

Stroke	Stroke characterisation^±^	Prestroke risk reduction strategies^±^	Poststroke risk reduction strategies^±^		

Cancer	Cancer prognosis and treatment^+^	Cancer screenings^#^	Cancer family history^±^		

Kidney disease	Kidney treatment status^#^	Kidney function assay^#^	Complete blood cell count		

Rare and developmental disorders	Biological parents and grandparents^±^	Developmental milestones^±^	Developmental disorders^±^	Rare disorders^±^	
	Birth history^±^	Neurodevelopmental assessment^±^	Epilepsy screener^#^		

Infectious diseases	Malaria exposure and treatment^±^	Trypanosomiasis signs and symptoms^+^	TB signs and symptoms^±^	HIV treatment^±^	Liver function assay^#^
	Malaria testing^#^	TB history^+^	Sexual behaviour^#^	CD4 count and percentage^#^	HIV signs and symptoms^+^
	Malaria signs and symptoms^+^	TB testing^#^	HIV exposure^±^	Quantitative viral load^#^	STDs^±^
	Trypanosomiasis testing^#^	Current TB^+^	HIV detection test^#^	Complete blood cell count^#^	STD signs and symptoms^+^

^*∗*^CVD: cardiovascular disease; HIV: human immunodeficiency virus; TB: tuberculosis; STD: sexually transmitted disease; ^+^adapted protocols; ^±^novel protocols; ^#^existing protocols.

## Data Availability

The data used to support the findings of this study are available from the corresponding author upon request. To request survey data, researchers can contact the first or second author.

## Supplementary Materials

Supplementary Materials Figure S1: overview of survey respondents by (A) country; (B) H3Africa Working Group; and (C) expertise.
