# Supplementary figures and images for: Developing Clinical Phenotype Data Collection Standards for Research in Africa
Source: Glob Health Epidemiol Genom. 2023 Sep 19;2023:6693323. doi: 10.1155/2023/6693323 (PMC10522421; doi:10.1155/2023/6693323)

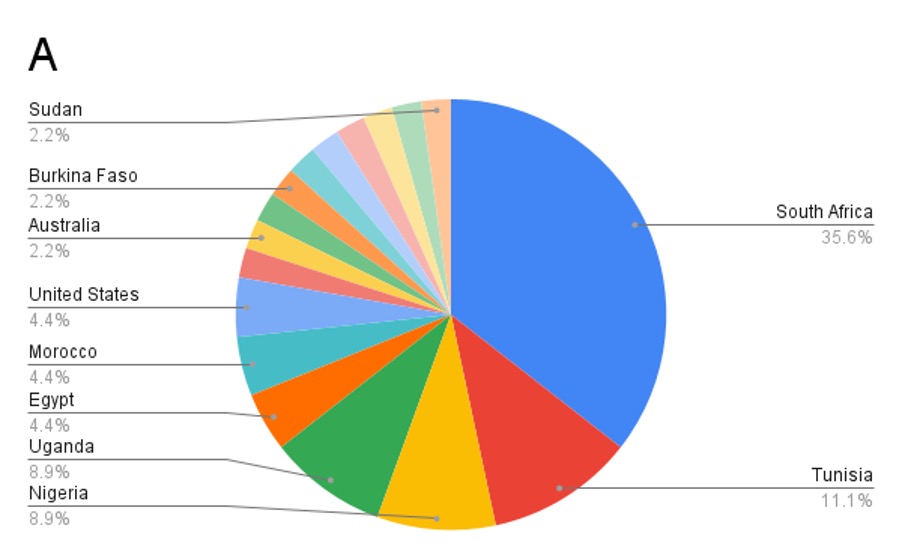

Supplement: Supplementary Materials — Figure S1: overview of survey respondents by (A) country; (B) H3Africa Working Group; and (C) expertise. [file 6693323.f1.zip › FigS1A.jpg]

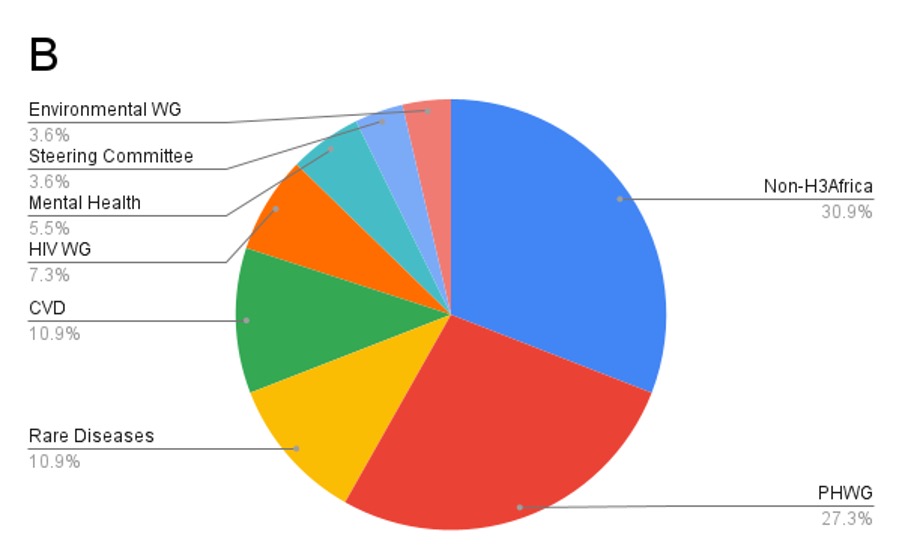

Supplement: Supplementary Materials — Figure S1: overview of survey respondents by (A) country; (B) H3Africa Working Group; and (C) expertise. [file 6693323.f1.zip › FigS1B.jpg]

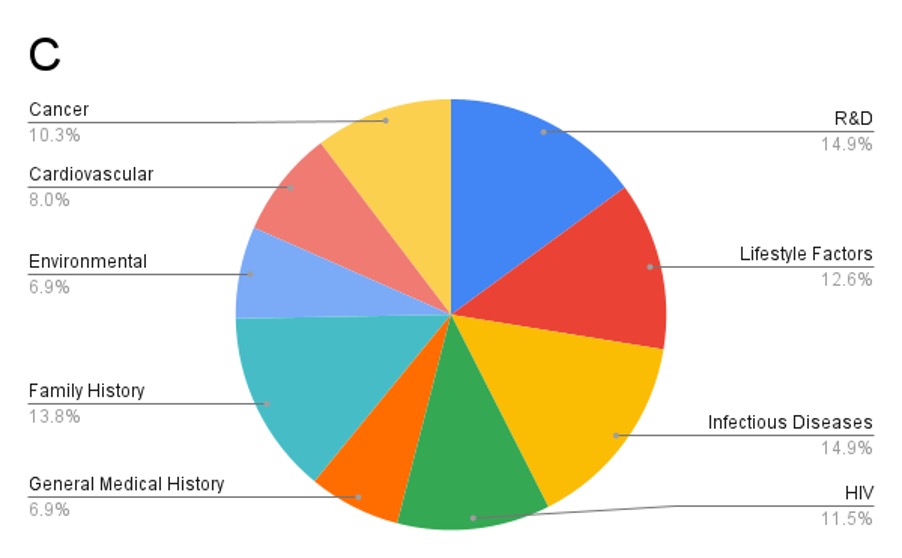

Supplement: Supplementary Materials — Figure S1: overview of survey respondents by (A) country; (B) H3Africa Working Group; and (C) expertise. [file 6693323.f1.zip › FigS1C.jpg]
